# Single-injection hyaluronic acid treatment demonstrates non-inferiority in the relief of symptomatic knee osteoarthritis: A randomized double-blind, multi-center controlled study

**DOI:** 10.1016/j.ocarto.2026.100752

**Published:** 2026-02-05

**Authors:** C. Ruosi, U.G. Longo, N. Giordan, L. Saracino, M.A. Minetto, C. Busso, N. Migliaccio, C. Melchior, A. Guardoli, E. Vaienti, G. Scita, F. Atzori, G. Chitoni, N. Orabona, F. Benazzo

**Affiliations:** a AOU Azienda Ospedaliera Federico II, Ortopedia e Traumatologia, Napoli, Italy; b Fondazione Policlinico Universitario Campus Bio-Medico, Roma, Italy; c Università di Torino, Dipartimento di Scienze Chirurgiche, Torino, Italy; d A.O. Cardarelli Napoli, Italy; e Ospedale di Conegliano, TV, Italy; f Ospedale S. Maria Borgo Val di Taro, PR, Italy; g Azienda Ospedaliero- Universitaria di Parma, Italy; h Presidio Sanitario Ospedale Cottolengo Torino, Italy; i Azienda Socio Sanitaria Territoriale Valcamonica, Esine BS, Italy; j Ospedale del Mare Napoli, Italy; k Fondazione Poliambulanza, Brescia, Italy; l Fidia Farmaceutici S.p.A, Abano Terme, Italy

**Keywords:** Osteoarthritis, Knee, Hyaluronic acid

## Abstract

**Objective:**

To compare two sodium hyaluronate formulations (HYMOVIS® ONE and MONOVISC®) for single intra-articular (IA) injection in patients with knee osteoarthritis pain.

**Methods:**

This randomized, controlled study included 347 subjects allocated to two treatment arms: HYMOVIS® ONE (n = 175) and MONOVISC® (n = 172). The primary endpoint assessed non-inferiority of HYMOVIS® ONE versus MONOVISC® based on change from baseline in the WOMAC LK3.1 A1 Pain subscale. Secondary outcomes evaluated patient response using OMERACT-OARSI criteria, changes in WOMAC stiffness and function scores, health-related quality of life (SF-12), global assessments by patients and clinicians, and rescue medication use.

**Results:**

The mean WOMAC LK3.1 A1 walking pain subscale score decreased significantly from baseline at week 12 in both groups, with reductions of 65 % for HYMOVIS® ONE and 66 % for MONOVISC®. At week 26, reductions were 73 % for HYMOVIS® ONE and 69 % for MONOVISC®. The non-inferiority test yielded a p-value of 0.0003. Clinically significant change from baseline was observed for all secondary endpoints. For the WOMAC LK3.1 Function subscale, the comparison between groups at week 26 showed a statistically significant difference (p = 0.0367) favoring HYMOVIS® ONE. Both treatments were well tolerated.

**Conclusions:**

A single injection of HYMOVIS® ONE was well tolerated and non-inferior to MONOVISC®, with improvements across all study endpoints maintained through 26 weeks, indicating sustained symptomatic relief of knee OA.

**Trial registration number:**

NCT06528600 (www.clinicaltrials.gov).

## 1 Introduction

Knee osteoarthritis (OA) is a prevalent and disabling condition, with a lifetime risk of symptomatic knee OA estimated at 44.7 % in the U.S. population [1]. It involves progressive degeneration of cartilage, subchondral bone, and synovium, leading to pain, functional impairment, and reduced quality of life. The absence of disease-modifying osteoarthritis (DMOADs) remains a major unmet need [2,3] and current management focuses on symptom relief, particularly pain, which drives disabilities and comorbidities [4].

Non-pharmacological interventions as education [5,6], weight loss [3,7], and an exercise program (i.e. aerobic, strengthening, or resistance exercises) [[5], [6], [7], [8], [9]], are first-line strategies but often insufficient for sustained symptom control. Pharmacological options, including paracetamol and NSAIDs, provide limited analgesia and carry significant gastrointestinal [10,11] and cardiovascular risks [12,13].

Consequently, IA therapies such as corticosteroids [14] and hyaluronic acid (HA) are considered when conservative measures fail. HA is a key component of healthy synovial fluid, essential for lubrication and shock absorption. In OA, HA concentration and molecular weight (MW) decrease [15,16] impairing joint function. Viscosupplementation with IA-HA injections aims to restore synovial fluid's viscoelasticity, reduce pain, and improve joint function, though evidence remains mixed [17]. Compared to corticosteroids, HA offers longer-lasting benefits and favorable safety profile [1], particularly in elderly or NSAID-sensitive patients, and may exert potential protective effects on joint structure [14,18].

Viscosupplementation HA-based products differ in molecular weight, cross-linking, and rheological properties, influencing efficacy and residence time [19]. Since HA is subject to degradation within the joint, strategies to prolong its persistence have become essential. Therefore, to increase residence time, several HA-based products have been developed. Among these, HYMOVIS® ONE (Fidia Farmaceutici S.p.A, Abano Terme, Italy) is a new non-avian sourced (bacterial fermentation) viscoelastic hydrogel that is not cross-linked, derived from the hexadecylamine-modified HA derivative HYADD® [20,21] and was recently approved by the U.S. FDA (PMA 150010/S005) [22]. Its network, stabilized by hydrophobic interactions from grafted alkyl side-chains [23], exhibits rapid structural recovery following deformation at physiological frequencies (e.g., walking or running) [24]. Critically, HYMOVIS® ONE uniquely demonstrates recovery of viscoelastic properties after repeated mechanical stress cycles [23] improving synovial fluid viscoelasticity and enhancing lubrication and shock absorption capabilities [20,23].

Moreover, previous studies have shown its effectiveness and tolerability in knee OA after a single injection [21,25,26].

This investigation aims to compare the efficacy and safety of a single IA injection of HYMOVIS®ONE (32 mg/4 mL) with MONOVISC® (80 mg/4 mL), a cross-linked HA-based product, with similar viscoelastic properties. We hypothesize that HYMOVIS® ONE is non-inferior to MONOVISC® in improving pain and joint function in patients with knee OA.

## 2 Methods

### 2.1 Study setting and ethical considerations

This was a Randomized, Double-Blin, Active Controlled, Multi-Center, Non-Inferiority Study performed in patients with knee OA. The study was conducted at 18 sites in Italy. The clinical investigation was conducted in compliance with ISO 14155, ICH Good Clinical Practice, the Declaration of Helsinki, EU Directive 93/42/EEC (as amended), MEDDEV guidelines, and applicable local regulations. The Clinical Investigation Plan (CIP), informed consent form, and related documents were approved by the Independent Ethics Committee (IEC) at each participating site and by the relevant National Regulatory Authorities prior to study initiation.

Written informed consent was obtained from all participants before enrollment. Patients were informed about the voluntary nature of participation, the right to withdraw at any time without consequences, and the potential risks and benefits of the study. Adequate time was provided for consideration, and insurance coverage details were explained. The study is prospectively registered at clinicaltrials.gov (NCT06528600).

### 2.2 Patient enrollment

Male and female patients aged 40–75 with primary OA of the medial/lateral femorotibial compartment, a body mass index (BMI) between ≥20 and < 35 kg/m^2^, symptoms lasting for ≥3 months and unresponsive to conservative treatments were primarily considered for inclusion. Other inclusion criteria were: Tegner score [27] ≥3; Kellgren-Lawrence [28] (K-L) radiological grade 2–3 in the target knee pain intensity in the target knee of 2–3 (WOMAC LK3.1 A1 Pain subscale) and no pain in the contralateral knee; willingness to discontinue oral/topical analgesics/NSAIDs (only paracetamol permitted). Subjects were excluded if affected by secondary knee OA, acute fractures, severe bone loss (e.g., advanced osteoporosis), or avascular necrosis affecting the target knee. Additional exclusion criteria included severe knee deformity, a history of knee replacement, arthroplasty, or recent arthroscopy (within the past year). Patients who had undergone osteotomy or any prior surgical intervention on either knee were also excluded, as were those with acute or recurrent synovitis or any musculoskeletal condition that could interfere with knee function assessment (e.g., neuromuscular disorders, severe ligament instability).

### 2.3 Data collection

The primary objective was to establish that HYMOVIS® ONE was not inferior to MONOVISC® in the Western Ontario and McMaster Universities Osteoarthritis Index (WOMAC) LK3.1 A1 Pain subscale [24] (walking on a flat surface) score change from baseline (CFB) at Week 12. HYMOVIS® ONE was considered non-inferior to MONOVISC® if the upper limit of the 95 % Confidence Interval (CI) of the difference of least squares means difference between the two treatment groups was lower than 0.32 at week 12. The WOMAC index (Likert format, version 3.1) assessed at 4, 12 and 26 weeks. Patients’ assessment of global status (PGA) and Clinical Observer Global Assessment (COGA) were recorded on a 0–100- points VAS at 4, 12 and 26 weeks.

Patients were classified as an OMERACT-OARSI [29] responder if: (1) improvement in pain or physical function ≥50 % and an absolute change ≥20; or (2) improvement of ≥20 % with an absolute change of ≥10 in at least two of the following three categories: pain, physical function, and patient's global assessment. Finally, a specific paper diary was used for patients to record their daily rescue medication (paracetamol) use, noting date, time, and reason for each dose.

Moreover, secondary objectives included the proportion of response (based on OMERACT/OARSI criteria) [29], the CFB in WOMAC LK3.1 Pain, Stiffness and Function subscales, the CFB in quality of life (based on the SF-12 questionnaire), the Patient Global Assessment (PGA) of disease activity and the Clinical Observer Global Assessment (COGA) of disease severity (based on Visual Analogue Scale), and the use of rescue medication, which have been evaluated up to 26 weeks. Furthermore, summary of treatment-emergent adverse events (TEAEs) was presented by treatment group.

### 2.4 Procedures and interventions

**Fig. 1:**
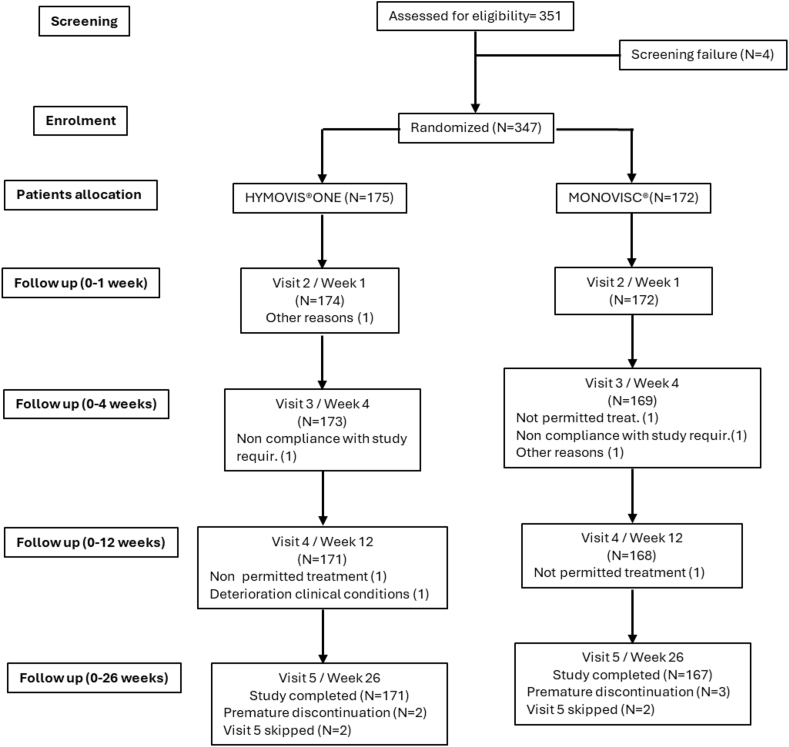
Patients disposition.

The investigation included a two-week screening phase (Day −14 to Day −1), a baseline/single treatment visit (Visit [V] 1, Day 0), and a 26-week follow-up evaluation phase (Fig. 1). To preserve the double-blind design despite visual differences between products, two site staff members had distinct roles: the Treating Physician (unblinded) administered the injection, while the Evaluator (blided) performed all efficacy and safety assessments. Patients remained blind to treatment allocation. During the treatment visit (V1, Day 0), the Treating Physician administered a single injection of HYMOVIS® ONE or MONOVISC®, if necessary, utilizing an echo-guided technique, of the product allocated by randomization.

### 2.5 Randomization

Eligible patients were randomized in a 1:1 ratio to receive a single intra-articular injection of HYMOVIS® ONE or MONOVISC®, using a validated software-generated randomization list. Each patient was assigned a unique randomization number linked to treatment kits.

Device accountability was managed by an independent monitor, separate from study monitors, without revealing treatment details.

### 2.6 Statistical methods

All statistical analyses were performed using SAS System Software (release 9.4), using a two-sided approach, unless for the non-inferiority analysis, and were considered significant when α was less than 0.05 (p < 0.05). Among the populations considered for the analysis, Intent-to-Treat (ITT) population included all patients signed the IC form and randomized to be treated; on the other hand, Per-Protocol (PP) population included all patients of the ITT completing Week 12 follow–up without any major protocol deviation. Safety Population (SAF) included all patients that signed the ICF and received the injection of investigational treatment.

#### 2.6.1 Primary performance analysis

The analysis of the primary performance endpoint was performed. An analysis of covariance (ANCOVA) model was used to analyze the primary endpoint. To test the non-inferiority a 95 % CI was computed on the ANCOVA-adjusted CFB mean difference at Week 12. If the upper limit of the 95 % CI of the difference of least squares means (LSM) difference of Test and Reference at Week 12 was lower than 0.32, then HYMOVIS® ONE was declared non-inferior to the comparator.

#### 2.6.2 Secondary performance analysis

The analysis of secondary performance endpoints was performed on the ITT population only. The analysis of the other continuous secondary performance endpoints was performed using a mixed model for repeated measures (MMRM) with the same approach used for the primary performance endpoint. The number and percentage of patients showing improvement in SF-12 scores and PGA (≥30 %, ≥40 %, or ≥50 %) were reported by treatment/visit. Rescue medication use was compared between treatment groups using a Chi-square test, and the total amount of rescue medication taken was analyzed using an ANCOVA model.

#### 2.6.3 Sample size estimation

The sample size calculation assumed a one-sided α = 0.025 level of significance, mean difference estimate of 0.10 (HYMOVIS® ONE arm better than the MONOVISC®) and a standard deviation of the CFB of 1.25. To achieve 80 % power, 141 subjects per treatment arm were needed to demonstrate that the mean CFB at Week 12 in the tested product arm was non-inferior to the comparator arm. Considering a drop-out rate of 20 %, 175 patients per arm were planned.

## 3 Results

### 3.1 Demographics

A total number of 347 patients were enrolled in the investigation. The first patient was enrolled on September 16th, 2020, and the last patient visit was completed on June 21st, 2024. Patients were randomized to the assigned treatment group: 175 were treated with HYMOVIS® ONE and 172 with MONOVISC® (Fig. 1). The demographic and baseline characteristics of the subjects were well balanced for sex, age, and symptom severity at baseline (Table 1). Mean score of WOMAC LK3.1 A1 Walking Pain subscale in the target knee at the baseline visit was comparable in the two treatment groups (2.1 in the test group and 2.2 in the other group) (Table 1).

**Table 1:** Demographic characteristics and baseline characteristics of patients.

	Statistic	HYMOVIS® ONE	MONOVISC®
		(N = 175)	(N = 172)
Age (years)	n	175	172
	Mean (SD)	59.4 (8.68)	59.3 (8.38)
	Median	59.0	60.0
	Min/Max	40.0/78.0	40.0/75.0
	Q1/Q3	53.0/65.0	53.0/65.0
Sex			
Male	n (%)	79 (45.1 %)	89 (51.7 %)
Female	n (%)	96 (54.9 %)	83 (48.3 %)
Race			
Caucasian	n (%)	174 (99.4 %)	171 (99.4 %)
Asiatic	n (%)	0 (0.0 %)	0 (0.0 %)
Black	n (%)	0 (0.0 %)	1 (0.6 %)
Other	n (%)	1 (0.6 %)	0 (0.0 %)
Height (m)	n	174	172
	Mean (SD)	1.70 (0.10)	1.70 (0.09)
	Median	1.69	1.70
	Min/Max	1.48/1.99	1.45/1.91
	Q1/Q3	1.63/1.76	1.65/1.76
Height (inches)	n	174	172
	Mean (SD)	66.80 (3.88)	67.08 (3.49)
	Median	66.34	66.93
	Min/Max	58.27/78.35	57.09/75.20
	Q1/Q3	64.17/69.29	64.96/69.29
BMI (kg/m^2^)	n	174	172
	Mean (SD)	26.31 (3.58)	26.58 (3.69)
	Median	25.75	26.11
	Min/Max	19.03/34.80	20.20/34.96
	Q1/Q3	23.66/28.57	24.06/29.10

### 3.2 Efficacy results

#### 3.2.1 Primary performance objective

**Fig. 2:**
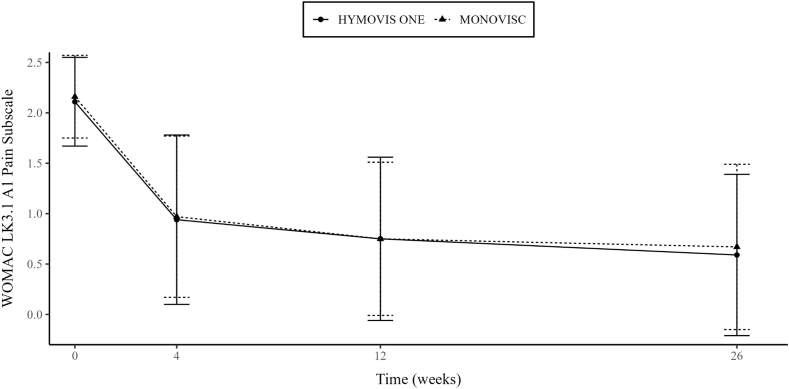
**WOMAC LK3.1 Walking pain subscore (A1) (±standard error) for each visit using roll back and LOCF imputation/ITT. Differences between HYMOVIS® ONE and MONOVISC® at baseline,4,12 and 26 weeks (HYMOVIS® ONE was confirmed to be non-inferior to MONOVISC® at week 12 (p = 0.0003 in the test of non-inferiority). Pain reduction and non-inferiority is maintained and confirmed also at week 26.**

The primary performance objective was achieved, as mean CFB WOMAC LK3.1 A1 Pain subscale score (walking on flat surface) decreased significantly (p < 0.001) in both treatment groups (Fig. 2) at week 12. The mean score reduction based on the multiple imputation analysis was similar in the two treatment groups (−1.38 in the HYMOVIS® ONE group and −1.42 in the MONOVISC® group). The adjusted mean difference between groups was 0.026 (95%CI, −0.1321 to 0.1836). As the upper limit of the 95 % CI of the difference of the LSM between the two treatment groups at week 12 was lower than the predefined limit of 0.32, HYMOVIS® ONE was demonstrated to be non-inferior to the comparator (p = 0.0003 in the test for non-inferiority). Notably, this non-inferiority margin was considerably lower than the Minimum Clinically Important Difference (MCID), which is approximately 0.7–0.8 points based on established literature. This more stringent threshold ensured that any observed differences below 0.32 would fall well below what is typically perceived by patients as clinically meaningful. The same findings were observed in all the analysis performed (roll back, LOCF, PP population), with no statistically significant differences between treatment groups, further confirming the non-inferiority of HYMOVIS® ONE in the primary effectiveness endpoint.

No statistically significant treatment-site effect was observed (p = 0.5669). Moreover, no important differences by gender and by age range were observed in primary effectiveness endpoint.

Secondary analysis of mean score of WOMAC LK3.1 A1 walking pain subscale significantly decreased from baseline to any post-baseline time point (week 4, 12 and 26) in both treatment groups, thus showing pain relief achieved rapidly and sustained up to 26 weeks after treatment (Fig. 2). The comparison between groups in the ITT population did not show statistically significant differences at any post-baseline time point and in the overall investigation period. Similarly, analysis using the MMRM model confirmed the non-inferiority of HYMOVIS® ONE vs. MONOVISC® at week 12. The proportion of patients with a percentage reduction from baseline ≥30 %, ≥40 %, ≥50 % was comparable between the two treatment groups at any post-baseline time point across all WOMAC subscales (Fig. 4).

Notably, the comparison of CFB to week 26 of WOMAC LK3.1 A1 Walking Pain subscale, based on the ANCOVA model, confirmed that HYMOVIS® ONE was non-inferior to MONOVISC® (Fig. 2). Additionally, the results of CFB through week 26 of WOMAC LK3.1 A1 Walking Pain subscale in the PP population were consistent with those observed in the ITT population. The extend of mean reduction from baseline to all post-baseline time points of Walking Pain subscale were comparable between males and females, across all age class, and in both treatment groups.

#### 3.2.2 Secondary performance objectives

**Fig. 3:**
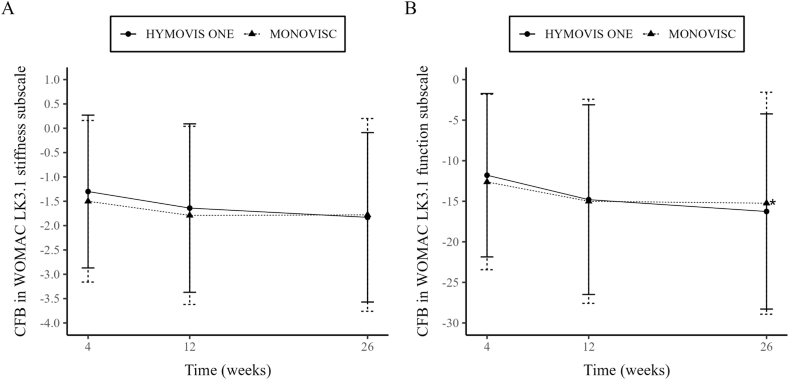
**A) Changes from baseline in WOMAC LK3.1 Stiffness subscale (±standard error) for each visit (ITT); B) Change from baseline in WOMAC LK3.1 Function subscale (±standard error) for each visit (ITT) ∗P = 0.0367. Comparison between treatments**.

Results for the remaining secondary effectiveness variables over week 26 were similar, with HYMOVIS® ONE eliciting comparable improvements to MONOVISC®. Regarding function, the comparison between groups showed that e adjusted mean difference was −0.150 (95 % CI, −2.2549 to 1.9540) at week 4, -0.921 (95 % CI, −3.0386 to 1.1967) at week 12, -2.244 (95 % CI, −4.3487 to - 0.1384) at week 26, and -1.105 (95 % CI, −2.3267 to 0.1167) in the overall investigation period (Fig. 3B). The comparison between groups did not show statistically significant differences at week 4 and week 12, however a statistically significant difference in favor of the HYMOVIS® ONE group was observed in WOMAC Function (physical function) scores at week 26 (p = 0.0367), (Fig. 3B). The mean reduction in stiffness from baseline at all post-baseline time points was comparable between treatment groups. The adjusted mean difference in the WOMAC LK3.1 A1 Stiffness subscale between HYMOVIS® ONE and MONOVISC® was 0.051 (95 % CI, −0.2456 to 0.3470) at week 4, -0.033 (95 % CI, −0.3310 to 0.2654) at week 12, -0.219 (95 % CI, −0.5157 to 0.0770) at week 26, and -0.067 (95 % CI, −0.2391 to 0.1048) in the overall investigation period. No statistically significant differences were found at any post-baseline time point (p = 0.7371 at week 4, p = 0.8290 at week 12, p = 0.1467 at week 26 and p = 0.4436 (Fig. 3A).

**Fig. 4:**
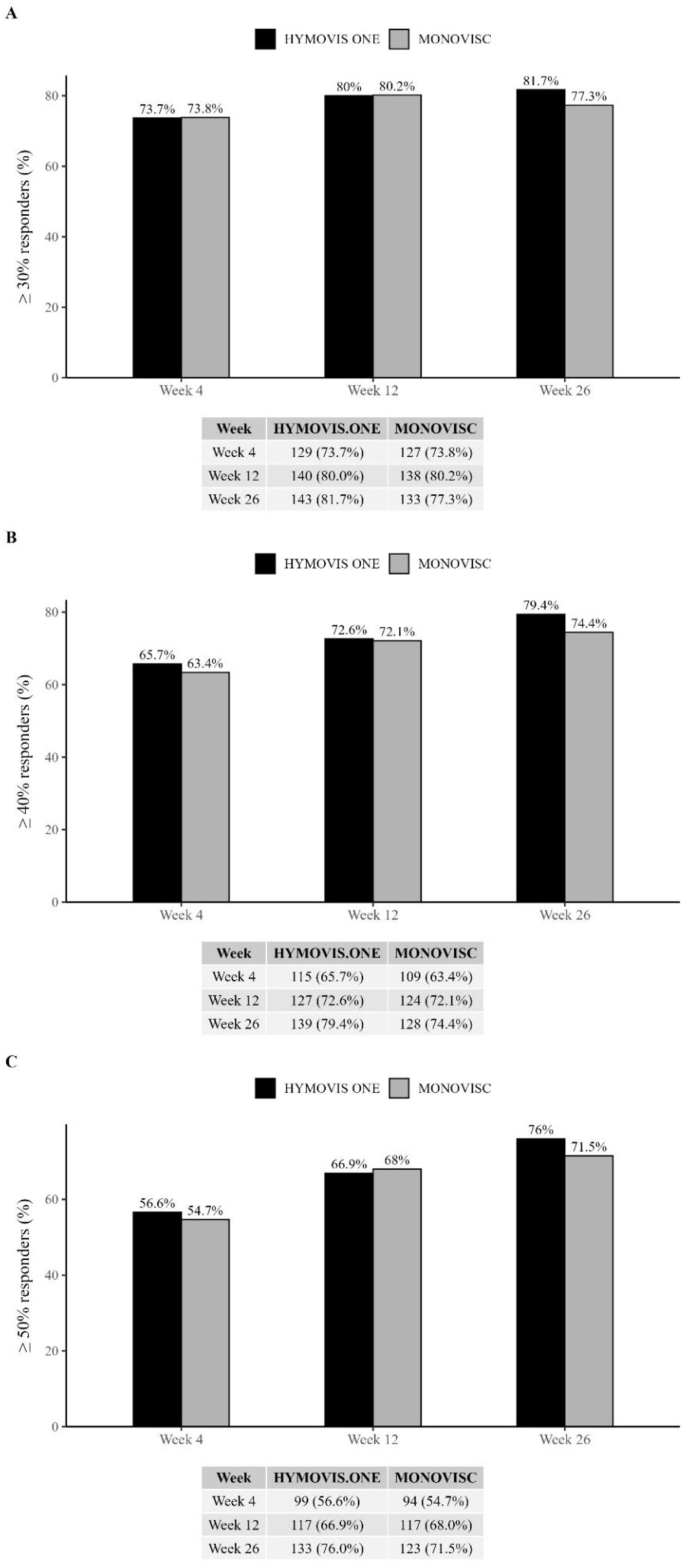
**A) Percentage of patients achieving a percentage improvement (reduction) compared to baseline of 30 %, inPain WOMAC LK3.1 subscales (ITT). Differences between HYMOVIS® ONE and MONOVISC® at 4,12 and 26 weeks.B) Percentage of patients achieving a percentage improvement (reduction) compared to baseline of 40 %, inPain WOMAC LK3.1 subscales (ITT). Differences between HYMOVIS® ONE and MONOVISC® at 4,12 and 26 weeks. C) Percentage of patients achieving a percentage improvement (reduction) compared to baseline of 50 %, in Pain WOMAC LK3.1 subscales (ITT). Differences between HYMOVIS® ONE and MONOVISC® at 4,12 and 26 weeks (Percentage are calculated relative to the total number of patients performing the visit in the ITT set by treatment group).**

The proportion of patients with a percent reduction from baseline of ≥30 % (Fig. 4A), ≥40 % (Fig. 4B) and ≥50 % (Fig. 4C) was comparable in the two treatments groups, at all post-baseline time points for all subscales.

A high proportion of patients in both treatment groups achieved success (based on OMERACT- OARSI Assessment) at both week 12 (86.9 % in the HYMOVIS® ONE group and 81.4 % in the MONOVISC® group) and week 26 (86.3 % in the HYMOVIS® ONE group and 85.5 % in the MONOVISC® group) (Table 2). The difference between groups was not statistically significant at both weeks 12 and 26. Similar response rates were observed in the two groups at week 12 and 26 for the first OMERACT-OARSI criterion (improvement in pain or physical function ≥50 % and an absolute change ≥20 %) (Table 3A). In contrast, the response rate for the second OMERACT-OARSI criterion (improvement of ≥20 % with an absolute change of ≥10 in at least two of the following three categories as pain, physical function, and patient's global assessment) was higher in the HYMOVIS® ONE group at week 12 (Table 3B). Moreover, in both treatment groups, patients with the following symptoms at baseline as swelling, tenderness, crepitus, inflammation, pain in motion, effusion and positive heel to buttock showed a trend toward resolution over the course of the study. At baseline, only a few patients in both treatment groups exhibited redness (and none had presence of redness at week 26) and patellar ballottement.

**Table 2:** Number and percentage of patients’ success at week 12 and 26, comparison between HYMOVIS® ONE and MONOVISC® (ITT) (percentages are calculated relative to the total number of patients performing the visit in the ITT set by treatment group). “Patients success” is defined by OMERACT-OARSI criteria (first and second).

	HYMOVIS® ONE		MONOVISC®	
	Patients	%	Patients	%
**Patients success at week 12**				
No	23	(13.1 %)	32	(18.6 %)
Yes	152	(86.9 %)	140	(81.4 %)
**Patients success at week 26**				
No	24	(13.7 %)	25	(14.5 %)
Yes	151	(86.3 %)	147	(85.5 %)

**Table 3:** A) Number and percentage of patients responder to the first OMERACT-OARSI criteria (high improvement in pain or in function ≥50 % and an absolute change ≥20) at week 12 and 26 (percentages are calculated relative to the total number of patients performing the visit in the ITT set by group treatment; B) Number and percentage of patients not responding to the second OMERACT- OARSI criteria (improvement in at least 2 of the 32 following: pain ≥20 % and absolute change ≥10; function ≥20 % and an absolute change ≥10; patient's global assessment ≥20 % and absolute change ≥10) at week 12 (#Table includes patients not responding at week 12 to the first OMERACT-OARSI set of responder criteria. The percentages are calculated on the number of patients not responding to the first OMERACT-OARSI set of responder criteria at week 12 in the ITT set by treatment group) and 26 (@Table includes patients not responding at week 26 to the first OMERACT-OARSI set of responder criteria. The percentages are calculated on the number of patients not responding to the first OMERACT-OARSI set of responder criteria at week 26 in the ITT set by treatment group).

**A)**				
	HYMOVIS® ONE		MONOVISC®	
	Patients (n)	%	Patients (n)	%
**High improvement at week 12**				
No	52	(29.7 %)	49	(28.5 %)
Yes	123	(70.3 %)	123	(71.5 %)
**High improvement at week 26**				
No	35	(20.0 %)	44	(25.6 %)
Yes	140	(80.0 %)	128	(74.4 %)

**B)**				

	HYMOVIS® ONE		MONOVISC®	

Patients (n)	%	Patients (n)	%
**High improvement at week 12#**				
No	23	(29.7 %)	32	(28.5 %)
Yes	29	(70.3 %)	17	(71.5 %)
**High improvement at week 26@**				
No	24	(20.0 %)	25	(25.6 %)
Yes	11	(80.0 %)	19	(74.4 %)

The results of quality of life, PGA and COGA showed an improvement in symptomatology to any post-baseline time-point in both treatment groups. There were no significant differences between groups in the use of rescue medication.

### 3.3 Safety: analysis of adverse events

In the HYMOVIS® ONE group, 51 patients (29.1 %) reported 219 Treatment-Emergent Adverse Event (TEAEs), while 58 patients (33.7 %) in MONOVISC® reported 348 TEAEs. No serious TEAEs or study discontinuations occurred. Adverse Device Event (ADEs) occurred in 3 (1.7 %) HYMOVIS® ONE patients (4 events) and 5 (2.9 %) MONOVISC® patients (9 events). Fewer TEAEs were reported in the 70–80 years age group for HYMOVIS® ONE, while slightly higher TEAEs rates were observed in the 50–60 years age group for the comparator. TEAEs of severe intensity were reported in one patient for each group (0.6 %): plantar fasciitis and influenza respectively in HYMOVIS® ONE and MONOVISC® group. All the other TEAEs in both treatment groups were of mild or moderate intensity. Adverse Events related to the product or to the injection procedure are summarized in Table 4.

**Table 4:** Number and percentage of adverse events (AEs) related to the product or injection procedure.

	HYMOVIS® ONE		MONOVISC®	
	Patients	%	Patients	%
Arthralgia	9	5.1	15	8.6
Joint contracture	0	0.6	1	0.6
Joint effusion	1	0.6	0	/
Joint swelling	1	0.6	0	/
Ligament pain	1	0.6	0	/
Metatarsalgia	1	0.6	0	/

## 4 Discussion

The present randomized, double-blid, controlled, multi-center clinical investigation evaluated the non-inferiority of a single IA injection of HYMOVIS® ONE versus MONOVISC® in 347 patients with knee OA. Both treatments demonstrated comparable efficacy in pain improvement over 12 and 26 weeks, with baseline demographics, disease characteristics, and clinical profiles well balanced between groups. The primary endpoint, change from baseline (CFB) in WOMAC LK3.1 A1 Walking Pain subscale scores at week 12, confirmed non-inferiority of HYMOVIS® ONE across all analytical methods in both the intention-to-treat (ITT) and per-protocol (PP) populations. Importantly, the non-inferiority of HYMOVIS® ONE versus MONOVISC® was sustained at week 26, as evidenced by the least squares mean difference in change from baseline (CFB) for the WOMAC LK3.1 A1 Walking Pain subscale using an ANCOVA model (95 % confidence interval within the pre-specified non-inferiority margin). The conclusion that HYMOVIS® ONE is non-inferior to MONOVISC® is robust, as all outcomes evaluated have been confirmed at each time point in all treatment groups. These findings are consistent with previous evidence supporting viscosupplementation as an effective option for symptomatic knee OA, particularly in patients with mild-to-moderate diseases who have not responded adequately to first-line conservative measures [30,31]. Compared to corticosteroids injections, which offer short-term pain relief (2–4 weeks) and may accelerate cartilage loss with repeated use [14], IA-HA injections provide sustained benefits up to 6 months and exhibit favorable safety profile [16,32]. On the other hand, oral NSAIDs, while effective for pain, are associated with significant gastrointestinal and cardiovascular risks [12,13], reinforcing the use of IA-HA as a safer alternative for long-term symptom management.

Our results align with previous investigations evaluating single-injection HA formulations. Petterson [33] reported significant improvements in WOMAC pain and function scores at 26 weeks with MONOVISC®, while Pavelka et al. (2020) demonstrated similar benefits with Synvisc-One® showing comparable WOMAL LK3.1 A1 score reduction versus baseline [34]. In the same study, which evaluated different doses of HYADD4, the main component of HYMOVIS® ONE, the 32 mg dosage, corresponding to that used in HYMOVIS® ONE, showed the greatest absolute improvement [34].

The current study adds novel evidence by demonstrating that HYMOVIS® ONE, a non-crosslinked hexadecylamine-modified HA hydrogel, achieves comparable outcomes to a crosslinked HA product, despite chemical differences. This suggest that reversible hydrophobic interactions and rapid viscoelastic recovery under mechanical stress may contribute to prolonged clinical efficacy [20,35].

Interestingly, the statistically significant improvement in WOMAC function scores at 26 weeks in favor of HYMOVIS® ONE may indicate an additional benefit restoring joint biomechanics, potentially linked to its unique rheological properties [21]. Functional improvement is a critical determinant of patient quality of life and long-term disease management, warranting further investigation.

When compared to alternative intra-articular orthobiologic therapies a broader category that includes PRP (Platelet-Rich Plasma), MSC (Mesenchymal Stem Cells), and other biological products, HA injections remain the most widely endorsed option in international guidelines due to their reproducible efficacy and safety [36,37]. While PRP has shown promise in some studies, result are heterogeneous and protocols lack of standardization [38,39]. Similarly, regenerative approaches (e.g., MSC) are still experimental and not recommended in routine practice [40]. Therefore, viscosupplementation continues to represent a well-established, evidence-based intervention for knee OA, offering a favorable benefit-risk profile.

The demonstrated non-inferiority of HYMOVIS® ONE to MONOVISC®, combined with its distinctive rheological properties and potential functional advantage, supports its inclusion among effective single-injection options. Future research should explore whether these structural differences translate into superior long-term outcomes or reduced need for retreatment compared to other HA formulations.

Remarkably, in this study, a single injection of HYMOVIS® ONE was demonstrated to be non-inferior to MONOVISC® in improving knee OA symptoms over 26 weeks, with both treatments showing comparable efficacy and safety profiles. While the precise mechanism of action of HA remains incompletely understood, the results support HYMOVIS® ONE as an effective option for symptomatic relief and functional improvement, without evidence to claim superiority or sustained benefits beyond the study period.

Moreover, HYMOVIS® ONE single intra-articular injection regimen provides clinically meaningful symptomatic relief and functional improvement in knee OA, combining therapeutic efficacy with enhanced patient compliance through reduced treatment burden, minimized procedural risks and patients’ discomfort compared to multi-injection regimens.

As previously mentioned, synovial HA in OA exhibits reduced concentration and MW. Effective viscosupplementation requires optimizing parameters beyond MW, including source, cross-linking, concentration, rheology, lubrication, and degradation resistance. An alternative to chemical cross-linking is grafting hydrophobic moieties onto HA. This enables reversible chain aggregation, enhancing properties and extending intra-articular residence without increasing HA MW or concentration [37]. Unlike covalent cross-linking, in HYMOVIS® ONE reversible hydrophobic interactions preserve critical viscoelasticity for synovial function while matching or exceeding cross-linked HA in rheological performance, degradation resistance, and cartilage lubrication [28,[38], [39], [40]]. Moreover, to the best of our knowledge HYMOVIS® ONE is a hydrogel and considered the only non-chemically cross-linked hyaluronan-based marketed viscosupplement showing gel-like behavior. Its *in vitro* characteristics are supported by clinical data, as demonstrated in the current investigation, which showed that HYMOVIS® ONE (a HYADD®4-based medical device) is not-inferior to the cross-linked medical device MONOVISC. Notably, as thoroughly discussed, the WOMAC LK3.1 Function subscale showed statistically superior outcomes for HYMOVIS® ONE versus the comparator at 26 weeks suggesting an additional potential long-term benefit in joint function recovery. To our knowledge, this is the first incidence of two single-injection viscosupplements being compared, where, HYMOVIS ONE®, demonstrated a statistically significant improvement in overall WOMAC function scores at 26 weeks over an active comparator hyaluronan viscosupplement, MONOVISC®.

In conclusion, this study proved that HYMOVIS® ONE is an effective and well-tolerated treatment for managing knee OA symptoms, demonstrating non-inferiority to MONOVISC®. Overall, the findings demonstrate that HYMOVIS® ONE and MONOVISC® exhibit comparable efficacy, safety, and tolerability profiles in the management of OA-related symptoms over 26 weeks.

## 5 Limitations

Several limitations should be acknowledged. First, the relatively short follow-up period (26 weeks) restricts conclusions regarding the long-term durability of treatment effects. Future studies with extended observation periods are needed to confirm sustained efficacy and structural benefits. Second, the absence of a placebo control group limits the ability to fully assess the magnitude of the treatment effect beyond active comparator performance. While the use of an FDA-approved HA product as an active comparator strengthens the non-inferiority design, it precludes evaluation of absolute efficacy versus placebo. Finally, the study did not include imaging or biomarker assessments, which could provide insights into potential disease-modifying effects of HA.

## Author contribution

Prof Benazzo: (1) Conception and design, (2) Final approval of the article Prof Ruosi: (1) Conception and design, (2) Final approval of the article.

Prof. Longo: (1) Research and Data Collection (2) Final approval of the article Dr Migliaccio: (1) Research and Data Collection (2) Final approval of the article Prof. Minetto: (1) Research and Data Collection (2) Final approval of the article Melchior: (1) Research and Data Collection (2) Final approval of the article.

Dr. Melchior: (1) Research and Data Collection (2) Final approval of the article Dr. Guardoli: (1) Research and Data Collection (2) Final approval of the article Prof. Vaienti: (1) Research and Data Collection (2) Final approval of the article Dr. Atzori: (1) Research and Data Collection (2) Final approval of the article Dr. Chitoni: (1) Research and Data Collection (2) Final approval of the article Dr. Orabona: (1) Research and Data Collection (2) Final approval of the article.

## Role of the funding source

The clinical trial NCT06528600 was promoted and funded by Fidia farmaceutici S.p.A, Italy. They fully financed the costs of the study and contributed to the design of the study, interpretation of data and in writing the manuscript.

## Conflict of interest

The authors declare that they have no conflict of interest. None of the above affected the way in which the results of this paper were analyzed and reported.
